# Clinical Relevance of Serum Klotho Concentration and Sagittal Abdominal Diameter

**DOI:** 10.3390/jcm11247376

**Published:** 2022-12-12

**Authors:** Jun-Wei Huang, Wen-Hui Fang, Wei-Liang Chen

**Affiliations:** 1 Division of Family Medicine, Department of Family and Community Medicine, Tri-Service General Hospital, School of Medicine, National Defense Medical Center, Taipei 114, Taiwan; 2 Division of Geriatric Medicine, Department of Family and Community Medicine, Tri-Service General Hospital, School of Medicine, National Defense Medical Center, Taipei 114, Taiwan; 3 Graduate Institute of Medical Sciences, National Defense Medical Center, Taipei 114, Taiwan

**Keywords:** serum Klotho concentration, sagittal abdominal diameter, visceral fat, insulin resistance

## Abstract

Klotho is an anti-aging gene. Studies have revealed its association with insulin resistance. Visceral fat is related to insulin resistance, and the sagittal abdominal diameter (SAD) can serve as a biomarker for visceral fat (VF). This study investigated the association between SAD and serum Klotho concentration (SKC). We enrolled 2301 participants from the 2011–2012 National Health and Nutrition Examination Survey (NHANES) dataset, and 49.2% of the enrolled individuals were male. Qualified participants were separated into four quartiles according to the SAD value. SKC values were obtained by ELISA. Demographic characteristics, body mass index (BMI), systolic blood pressure, and biochemistry parameters with significance were analyzed using multivariate linear regression models. The mean age of the study participants was 57.22 ± 10.53 years. The fully adjusted regression model showed a negative association between SAD and SKC (*p* < 0.05), with a β-coefficient of −12.02. We also analyzed subgroups of participants according to age and BMI. Participants with an age ≥65 and <65 years old were each negatively associated with SKC, and this association was significant for participants with a BMI ≥ 30 kg/m^2^ (*p* = 0.001, β-coefficient: −18.83). We also found a concentration-dependent relationship between SAD and SKC. In conclusion, VF and SKC are associated, and SAD can serve as a surrogate of VF and an indicator of SKC.

## 1. Introduction

Kuro-o et al. first discovered the Klotho gene in 1997 and identified a mutation that led to human-like premature aging in mice [1]. The Klotho gene has since been studied in detail. Evidence shows that its overexpression extends lifespan in animal models [2]. On the other hand, decreases in the serum Klotho concentration were found to be associated with aging and lead to age-related diseases, such as chronic kidney disease (CKD), cardiovascular disease (CVD), diabetes, and a shortened life expectancy [3]. Klotho proteins have three isoforms: α-, β-, and γ. All isoforms are single-pass transmembrane proteins [4]. They are part of the endocrine system that modulates several metabolic pathways in mammals. β -Klotho is predominantly expressed in the liver and adipose tissue. It regulates bile acid metabolism and synthesis by facilitating the binding of fibroblast growth factor (FGF) receptor 4 with FGF15 and FGF19. It also increase obesity resistance via the FGF21 pathway [5]. γ- Klotho is a promoter of certain cancers [6]. However, the word “Klotho” usually refers to α-Klotho, a receptor of FGF23 that maintains vitamin D and phosphate equilibrium. Kuro-o et al. also discovered that there are multiple forms of α-Klotho, including membrane, soluble, circulating, and secreted forms. The soluble form of the α-Klotho protein typically acts as an endocrine factor, and can be detected in blood, urine, and cerebrospinal fluid [7]. It interacts with a variety of target tissues and organs. Recent studies report that α-Klotho participates in energy metabolism. In 2012, α-Klotho was reported to control adipogenesis, a process in which differentiated preadipocytes become mature adipocytes. Klotho also interacts with adipogenic factors, such as peroxisome proliferator-activated receptor gamma and fatty acid-binding protein 4, suggesting that α-Klotho is associated with intracellular lipid accumulation. A mutated Klotho gene increases resistance to gaining body weight in a murine model [5]. Another study reported a relationship between Klotho and metabolic syndrome. Cheng et al. demonstrated that the soluble α-Klotho concentration is inversely related to abdominal fat and serum triglyceride (TG) concentrations. 

Obesity is a condition of excessive fat accumulation or abnormal distribution. According to the World Health Organization, a body mass index (BMI) ≥25 is considered overweight, and ≥30 is considered obese [7]. In 2016, approximate 650 million people worldwide were obese. The most common method used to evaluate obesity is BMI. However, BMI is insufficient for measuring visceral fat (VF) and its impact on the human body. Anthropometric measurements such as the sagittal abdominal diameter (SAD) are preferred because of their accessibility. SAD is an emerging surrogate measure for VF and is an effective tool for evaluating glucose metabolism [8], insulin resistance (IR) [9], and other conditions. Notably, both α-Klotho and VF are strongly related to IR [10,11]. A few past studies have addressed issues about Klotho and anthropometric parameters. One cross-sectional study published in 2019 revealed that BMI is positively associated with the plasma Klotho level, although the association was not significant after adjusting for the lean mass index. Landry et al. demonstrated that α-Klotho concentrations in cerebrospinal fluid have an inverse correlation with BMI. However, no study focuses on whether SAD could be a surrogate or predictor for Klotho. Our study aims to elucidate the relationship between SAD and Klotho.

## 2. Materials and Methods

### 2.1. Source and Participants

**Figure 1:**
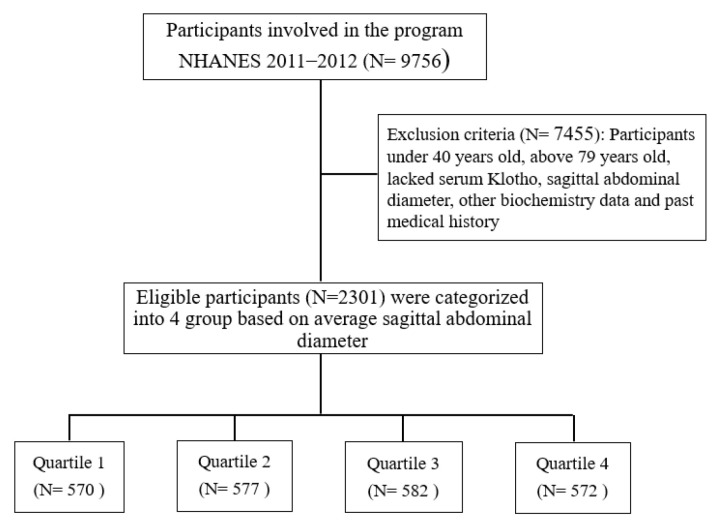
Study flowchart.

This study enrolled individuals between 40–79 years of age from the National Health and Nutrition Examination Survey (NHANES) dataset. The NHANES survey is a cross-sectional survey of the US population, and the data consist of interview questionnaires, psychological assessments, physical examinations, and biochemical tests. Datasets spanning two years are available for analysis, and we acquired the dataset from the 2011–2012 study cycle (Supplementary Materials), owing to limited data of SKC in NHANES 2011–2012. A total of 9756 respondents were screened, and participants with missing data, such as anthropometric parameters, serum Klotho, demographic features, and previous medical history, were excluded. Finally, 2301 participants met the inclusion and exclusion criteria, and their data were analyzed. Figure 1 shows the selection process.

### 2.2. Sagittal Abdominal Diameter (SAD)

SAD is an anthropometric parameter, so technicians in the Mobile Examination Center (MEC) used Holtain-Kahn abdominal calipers to measure the outboard range from the front of abdomen to the small of the back at the height of the iliac crest in supine position. The NHANES survey of 2011–2012 reported SAD values as the mean value of two measurements, although it also reported some means of up to four measurements for individuals exhibiting higher variability between SAD measurements [12]. Pregnant women and those weighing over 600 pounds were excluded.

### 2.3. Klotho Measurement

The data report on soluble α-Klotho, which serves as a paracrine and endocrine factor for various target organs. The NHANES lab acquired pristine serum specimens that were stored at −80 °C with frozen carbon dioxide until analysis. Klotho was measured with enzyme-linked immunosorbent assays (ELISA) manufactured by IBL International using a high-affinity antibody to the Klotho extracellular domain with selectivity. Klotho values represent the mean of two independent measurements of each sample. Two quality control specimens were also included in each ELISA plate to evaluate the assay’s linearity. Graphs of the expected and acquired values demonstrated good linearity in the measured range (R2 = 0.998 and 0.997, respectively). The coefficients of variation (CVs) for intra-assay precision for two recombinants were 3.2% and 3.9%, and, for two human samples, were 2.3% and 3.3%. The inter-assay CVs for the recombinant samples were 2.8% and 3.5%, and those of human samples were 3.8% and 3.4%. The analysis results were delivered to the Oracle Management System laboratory and evaluated. The measurements were repeated when duplicate sample values differed by >10%. When the measured value of the quality control sample exceeded two standard deviations (SDs) of the assigned value, the entire run was rejected and then repeated. Sensitivity was 6 pg/mL. The reference range of Klotho for healthy individuals is 285.8 to 1638.6 pg/mL, with an average of 698.0 pg/mL.

### 2.4. Covariates

This study included demographic features such as age, sex, and race/ethnicity. Personal and medical history were derived from self-reported questionnaire, including a history of smoking, congestive heart failure (CHF), coronary heart disease (CHD), and angina/angina pectoris. BMI was computed using the participant’s body weight in kilograms divided by the square of their height in meters (kg/m^2^). The biochemistry data were obtained via blood samples, and included aspartate transaminase (AST), TG, and fasting serum glucose. AST was derived via DxC800. It calculated the rate of change in absorbance at 340 nm as the activity of AST in a fixed-time interval. TG levels were estimated using a series of coupled enzyme reactions. TG was hydrolyzed to obtain glycerol and then oxidized by glycerol oxidase. Hydrogen peroxide (H_2_O_2_), one of the reaction products, was converted via peroxidase to a phenazone. Absorbance was estimated at 500 nm. Fasting glucose was obtained by the hexokinase method, which converts glucose into glucose-6-phosphate. Glucose-6-phosphate was converted into gluconate 6-phosphate in the presence of nicotinamide adenine dinucleotide phosphate (NADP+). The reduction in NADP+ increases absorbance at 340 nm (secondary wavelength = 700 nm), which was measured. Systolic blood pressure was taken after 5 min of resting in a seated posture. Three consecutive blood pressure measurements were obtained. Both systolic and diastolic blood pressures were measured in the mobile examination center (MEC). Subjects with abnormalities on both arms, such as rashes, casts, or edema, were excluded.

### 2.5. Statistical Analysis

Statistical Product and Service Solutions version 18.0 (SPSS Inc., Chicago, IL, USA) software was used to conduct the analysis. Continuous covariates were demonstrated as average values and SD. Discrete covariates were presented as numbers and percentages. Β-coefficients, 95% confidence intervals, and two-sided *p*-values were calculated using a one-way analysis of variance (ANOVA). *p*-values < 0.05 were regarded as significant. Multivariate regression analysis was used for serum Klotho concentration (SKC) and other variables. Model 1 was unadjusted, and Model 2 was adjusted for age, ethnicity, sex, and BMI. Model 3 was based on Model 2, with additional adjustment for systolic blood pressure, AST, TG, and fasting glucose. Model 4 was adjusted based on Model 3, with additional adjustments for a history of CHF, CHD, angina/angina pectoris, and smoking.

## 3. Results

Our study included 9756 participants from the NHANES dataset of the 2011–2012 cycle. A total of 2301 participants were enrolled. Data were lacking for the 7455 participants who were excluded. 

### 3.1. Study Population Demographics

The demographic features of the study population are presented in Table 1. The average age of the entire study group was 57.22 ± 10.53 years, and 49.2% of the participants were men. Qualified participants were separated into four quartiles based on SAD. Comparing lower quartiles with higher quartiles reveals that higher SAD quartiles tended toward a lower SKC, older age, higher level of TG, higher systolic blood pressure, higher BMI, and higher fasting serum glucose (*p* < 0.001). A higher prevalence of smoking, angina/angina pectoris, CHD, and CHF was also observed in higher quartiles (*p* < 0.001).

### 3.2. Association between SAD and SKC

We analyzed the relationship between SAD and SKC using a multivariate regression model (Table 2). The fully adjusted models exhibited a negative correlation (β-coefficient = −12.023) between SAD and SKC, with significance (*p* = 0.002).

Table 3 shown that, when eligible subjects were divided into two groups according to age (<65 years old and ≥65 years old), significantly negative correlations between SAD and SKC were found in both fully adjusted models (*p* = 0.048 and 0.004, β-coefficient were −8.500 and −24.269); however, the absolute value of the β-coefficient was higher in the older group than in the younger group.

When the study population was divided according to BMI, a cutoff value of 30.0 kg/m^2^ (Table 4) was used. Significantly negative associations were observed in the fully adjusted models for the high-BMI group (β-coefficient = −18.833; *p* = 0.001).

Because SKC decreased linearly in each quartile of enrolled participants, we conducted a multivariate analysis to investigate the trend between SKC and SAD. SKC and SAD demonstrated a negative association in the fully adjusted model for the highest SAD quartile (Table 5), which means that subjects in the higher SAD quartiles tended to have a significantly lower SKC (*p* for trend = 0.012). These findings indicate that there is a concentration-dependent relationship between SAD and SKC.

## 4. Discussion

Our study aims to elucidate the relationship between SAD and SKC. We found an inverse relationship between SAD and SKC using subjects registered in the NHANES database, a nationwide assessment of the health and nutritional status of the US population. When subjects were categorized by age, the inverse correlation was significant in both groups (<65 years old and, ≥65 years old). Subjects with a BMI in the obese range (≥30 kg/m^2^) were found to have a lower SKC. In conclusion, there is a concentration-dependent influence of SAD on SKC levels.

### 4.1. SAD and Chronic Disease

SAD, as measured by anthropometry, has been considered as a surrogate marker for VF [11,13]. Previous studies analyzed the relationship between VF and chronic diseases, including diabetes and CVD [14], and demonstrated that visceral adipose tissue generates high levels of proinflammatory cytokines, including interleukin-6 and tumor necrosis factor-alpha (TNF-α). Elevated cytokine levels subsequently lead to macrophage accumulation, vascular wall damage, and interference with insulin signaling [15]. 

### 4.2. Klotho and Age

Aging is considered to be a chronic process of inflammation [16] that causes gradual organ dysfunction. Klotho is considered to be an anti-aging gene. Several studies emphasized that mutations in or the knockout of the Klotho gene lead to premature aging in murines [1,2,17,18]. These murines experienced age-related diseases that shortened their lifespan, as they do in humans, such as osteoporosis, arteriosclerosis, and emphysema [4]. On the contrary, the overexpression of Klotho extends the lifespan. In 2003, researchers disclosed that Klotho functions as a peptide hormone that inhibits insulin and insulin-like growth factor 1 (IGF-1) signaling pathways, resulting in an extended lifespan [1]. These findings focus more on Klotho as a therapeutic strategy via the reactivation of related genes or exogenous Klotho supplements [4]. 

SKC is significantly related to age [19]. However, the age-specific reference concentration for Klotho is lacking. In our study, we chose 65 as a cutoff age; however, both groups (<65 and ≥65 years of age) showed a significant negative correlation between SAD and SKC. Traditionally, an age ≥ 65 is considered elderly, although the definition of elderly has been debated in recent years. The significance in both groups may be because aging is a continuous process without a specific starting point. In 2010, Yamazaki et al. reported that children SKCs were higher than adults SKCs [20].

### 4.3. Klotho and BMI

Evidence supporting obesity contributing to chronic inflammation is expanding. Obesity triggers proinflammatory cytokines and causes comorbidities such as diabetes and IR [21]. BMI is a preferred modality for the assessment of obesity [22], and numerous studies have confirmed the association between BMI and inflammation. In 2020, a cross-sectional study showed that the success of weight reduction interventions was strongly related to a decrease in inflammatory markers. The authors also stated that BMI was a reliable indicator of several anti-aging biomarkers [23]. Another cross-sectional study in 2018 demonstrated that Klotho was strongly associated with BMI in middle-aged adults. Furthermore, Amitani et al. found that Klotho indicates a normal nutritional condition and elicits SKC reductions in obesity subjects [24]. There are debates about the best tool for evaluating obesity and related comorbidities. BMI, waist circumference, and SAD are the most used measurements. BMI and body fat are not linearly correlated and differ by gender. Several recent articles suggest that SAD correlates better with cardiovascular and diabetes risk factors in men and women [8,25]. SAD is recommended due to its accessibility. SAD can be measured by a computed tomography scan or anthropometry, which only requires simple measurement tools.

### 4.4. Limitations

There are some limitations of our study. First, this study was a retrospective observational analysis of the NHANES database, a transverse survey. Therefore, the relationship between SAD and SKC may not be definitively established. Second, certain demographic traits were collected by self-reported questionnaires, including a history of smoking and medical history, and over-reporting bias may be inevitable. Third, there was a limited population regarding SAD. SKC was collected from individuals between 40–79 years old in our study. The connection between SKC and SAD for individuals <40 years old and >79 years old remained unclear.

## 5. Conclusions

Klotho is an emerging biomarker for longevity. Our research showed that SAD is inversely related to SKC, with concentration-dependent effects. Individuals older and younger than 65 years old each exhibited an inverse relationship between SAD and SKC. Obesity (BMI ≥ 30) was significantly associated with SKC. In conclusion, we reported that VF affects SKC, and that SAD can serve as a surrogate for visceral fat and as an indicator of SKC. Additional longitudinal studies are warranted to support our conclusions and to discover potential clinical uses of SAD in clinical conditions.

## Figures and Tables

**Table 1:** Characteristics of participants for each quartile of sagittal abdominal diameter.

Quartiles of Sagittal Abdominal Diameter						
Characteristics of participants	Q1 (<20.4 cm)	Q2 (20.4 to <23.2 cm)	Q3 (23.2 to <26.1 cm)	Q4 (>26.1 cm)	Total	*p*
	(*n* = 570)	(*n* = 577)	(*n* = 582)	(*n* = 572)	(*n* = 2301)	Value
Continuous Variables ^1^						
Klotho (pg/mL)	956.38 (303.37)	911.56 (316.43)	888.53 (324.32)	877.95 (310.98)	892.82 (316.62)	0.029
Age (years)	52.66 (9.54)	56.36 (10.31)	57.34 (10.65)	58.20 (10.49)	57.22 (10.53)	<0.001
BMI (kg/m^2^)	21.15 (2.03)	24.15 (2.39)	27.52 (2.87)	34.78 (6.05)	29.38 (6.42)	<0.001
Systolic blood pressure (mmhg)	118.77 (16.86)	123.58 (17.98)	128.07 (17.71)	129.36 (17.62)	127.14 (17.92)	<0.001
AST (U/L)	25.98 (19.51)	25.27 (14.13)	26.09 (16.64)	26.60 (17.04)	26.12 (16.49)	0.559
Triglyceride (mg/dL)	99.02 (56.08)	133.52 (97.08)	164.01 (132.74)	189.53 (152.30)	164.34 (134.28)	<0.001
Glucose, serum(mg/dL)	90.33 (18.31)	98.05 (34.44)	106.48 (46.16)	115.87 (47.52)	107.62 (44.13)	<0.001
Categorical Variables ^2^ (%)						
Male	27.3 (35)	39.2 (187)	54.0 (415)	53.3 (494)	49.2 (1131)	<0.001
Hispanic white	5.5 (7)	9.2 (44)	10.3 (79)	10.4 (96)	9.8 (226)	<0.001
History of smoking	35.9 (46)	42.3 (202)	45.0 (346)	52.1 (483)	46.8 (1077)	<0.001
Ever had angina/angina pectoris	0.8 (1)	0.6 (3)	2.6 (20)	4.7 (44)	3.0 (68)	<0.001
Ever had CHD	1.6 (2)	2.3 (11)	4.3 (33)	4.2 (39)	3.7 (85)	<0.001
Ever had CHF	0.0 (0)	1.9 (9)	2.6 (20)	5.6 (52)	3.5 (81)	<0.001

^1^ Values were expressed as mean (standard deviation). ^2^ Values in the categorical variables were expressed as % (numbers). Abbreviations: AST, aspartate aminotransferase; BMI, body mass index; CHD, coronary heart disease; CHF, congestive heart failure.

**Table 2:** Association between sagittal abdominal diameter and serum klotho concentration.

Models ^1^	β ^2^ (95% CI)	*p* Value
Model 1	−3.530 (−6.652, −0.408)	0.027
Model 2	−12.133 (−19.419, −4.846)	0.001
Model 3	−13.332 (−20.790, −5.875)	<0.001
Model 4	−12.023 (−19.517, −4.530)	0.002

^1^ Adjusted covariates: Model 1 = unadjusted; Model 2 = age, race, gender, BMI; Model 3 = Model 2 + systolic blood pressure, AST, TG, fasting glucose; Model 4 = Model 3 + history of CHF, CHD, angina/angina pectoris, and history of smoking. ^2^ β coefficients were interpreted as change in serum Klotho concentration for each increase in sagittal abdominal diameter. Abbreviation: AST, aspartate aminotransferase; BMI, body mass index; CHD, coronary heart disease; CHF, congestive heart failure; TG: triglyceride.

**Table 3:** Association between serum Klotho concentration and sagittal abdominal diameter with age difference.

Models ^1^	β ^2^ (95% CI)	*p* Value
	**Age < 65 y/o**	
Model 1	−2.937 (−6.382, 0.509)	0.095
Model 2	−8.823 (−16.998, −0.648)	0.034
Model 3	−10.013 (−18.408, −1.617)	0.019
Model 4	−8.500 (−16.913, −0.088)	0.048
	**Age ≥ 65 y/o**	
Model 1	−5.087 (−12.413, −2.239)	0.173
Model 2	−23.312 (−39.402, −7.221)	0.005
Model 3	−25.648 (−41.918, −9.378)	0.002
Model 4	−24.269 (−40.753, −7.784)	0.004

^1^ Adjusted covariates: Model 1 = unadjusted; Model 2 = age, race, gender, BMI; Model 3 = Model 2 + systolic blood pressure, AST, TG, fasting glucose; Model 4 = Model 3 + history of CHF, CHD, angina/angina pectoris, and history of smoking. ^2^ β coefficients were interpreted as change in serum Klotho concentration for each increase in sagittal abdominal diameter. Abbreviation: AST, aspartate aminotransferase; BMI, body mass index; CHD, coronary heart disease; CHF, congestive heart failure; TG: triglyceride.

**Table 4:** Association between serum klotho concentration and sagittal abdominal diameter with BMI difference.

Models ^1^	β ^2^ (95% CI)	*p* Value
	**BMI < 30**	
Model 1	−8.489 (−14.932, −2.045)	0.010
Model 2	−8.321 (−18.992, 2.350)	0.126
Model 3	−9.043 (−19.936, 1.851)	0.104
Model 4	−6.882 (−17.867, 4.104)	0.219
	**BMI ≥ 30**	
Model 1	−6.465 (−12.668, −0.262)	0.041
Model 2	−17.743 (−28.553, −6.933)	0.001
Model 3	−20.019 (−31.087, −8.951)	<0.001
Model 4	−18.833 (−29.974, −7.693)	0.001

^1^ Adjusted covariates: Model 1 = unadjusted; Model 2 = age, race, gender, BMI; Model 3 = Model 2 + systolic blood pressure, AST, TG, fasting glucose; Model 4 = Model 3 + history of CHF, CHD, angina/angina pectoris, and history of smoking. ^2^ β-coefficients were interpreted as change in serum Klotho concentration for each increase in sagittal abdominal diameter. Abbreviation: AST, aspartate aminotransferase; BMI, body mass index; CHD, coronary heart disease; CHF, congestive heart failure; TG: triglyceride.

**Table 5:** Association between the sagittal abdominal diameter comparison and serum Klotho concentration.

Models ^1^	Sagittal Abdominal Diameter Quartiles	β ^2^ (95% CI)	*p* Value	*p* for Trend
Model 1	Q2 vs. Q1	−31.265 (−94.542, 32.013)	0.333	0.006
	Q3 vs. Q1	59.022 (−119.705, 1.661)	0.057	
	Q4 vs. Q1	−69.761 (−129.646, −9.876)	0.022	
Model 2	Q2 vs. Q1	−23.642 (−87.926, 40.642)	0.471	0.010
	Q3 vs. Q1	−54.047 (−119.538, 11.445)	0.106	
	Q4 vs. Q1	−84.476 (−160.973, −7.979)	0.030	
Model 3	Q2 vs. Q1	−21.654 (−85.882, 42.514)	0.508	0.009
	Q3 vs. Q1	−52.145 (−117.984, 13.693)	0.121	
	Q4 vs. Q1	−86.078 (−163.302, −8.854)	0.029	
Model 4	Q2 vs. Q1	−18.312 (−82.319, 45.695)	0.575	0.012
	Q3 vs. Q1	−50.468 (−116.128, 15.191)	0.132	
	Q4 vs. Q1	−80.466 (−157.585, −3.348)	0.041	

^1^ Adjusted covariates: Model 1 = unadjusted; Model 2 = age, race, gender, BMI; Model 3 = Model 2 + systolic blood pressure, AST, TG, fasting glucose; Model 4 = Model 3 + history of CHF, CHD, angina/angina pectoris, and history of smoking. ^2^ β coefficients were interpreted as change in serum Klotho concentration for each increase in sagittal abdominal diameter. Abbreviation: AST, aspartate aminotransferase; BMI, body mass index; CHD, coronary heart disease; CHF, congestive heart failure; TG: triglyceride.

## Data Availability

The datasets used and analyzed during the current study are available from the corresponding author on reasonable request.

## Supplementary Materials

The study was based on NHANES 2011–2012 cycle. Supporting information could be downloaded at: https://wwwn.cdc.gov/nchs/nhanes/continuousnhanes/default.aspx?BeginYear=2011 (accessed on 19 November 2022).
